# Should Clinicians Split or Lump Psychiatric Symptoms? The Structure of Psychopathology in Two Large Pediatric Clinical Samples from England and Norway

**DOI:** 10.1007/s10578-017-0777-1

**Published:** 2017-12-14

**Authors:** Lorena Fernández de la Cruz, Pablo Vidal-Ribas, Nada Zahreddine, Børge Mathiassen, Per Håkan Brøndbo, Emily Simonoff, Robert Goodman, Argyris Stringaris

**Affiliations:** 1 Department of Clinical Neuroscience, Child and Adolescent Psychiatry Research Center, Karolinska Institutet, Gävlegatan 22 (Entré B), Floor 8, 11330 Stockholm, Sweden; 2 Department of Child and Adolescent Psychiatry, Institute of Psychiatry, Psychology & Neuroscience, King’s College London, London, UK; 3 Mood Brain & Development Unit, Emotion and Development Branch, National Institute of Mental Health, Bethesda, MD USA; 4 Department of Psychiatry, Saint-Joseph University, Beirut, Lebanon; 5 Regional Centre for Child and Youth Mental Health and Child Welfare – North, UiT The Arctic University of Norway, Tromsō, Norway

**Keywords:** Nosology, Factor structure, Construct validity, Predictive value, Strengths and Difficulties Questionnaire

## Abstract

It has been suggested that the structure of psychiatric phenomena can be reduced to a few symptom dimensions. These proposals, mainly based on epidemiological samples, may not apply to clinical populations. We tested the structure of psychiatric symptoms across two pediatric clinical samples from England (N = 8434) and Norway (N = 5866). Confirmatory factor analyses of the parent-reported Strengths and Difficulties Questionnaire (SDQ) evaluated the relative fit of several models, including a first-order model, a second-order model with the widely-established broad symptom dimensions of internalizing-externalizing, and two bi-factor models capturing a general psychopathology factor. Predictive value of the SDQ subscales for psychiatric disorders was examined. A first-order five-factor solution better fit the data. The expected SDQ subscale(s) related best to the corresponding psychiatric diagnosis. In pediatric clinical samples, a granular approach to psychiatric symptoms where several dimensions are considered seems to fit the data better than models based on lumping symptoms into internalizing/externalizing dimensions.

**Electronic supplementary material:**

The online version of this article (10.1007/s10578-017-0777-1) contains supplementary material, which is available to authorized users.

## Introduction

It has been suggested that the structure of psychiatric phenomena can be reduced to a few dimensions of symptoms. For example, Kendler et al. [1] assessed a cohort of more than 5600 adult twins from a population-based registry using DSM diagnostic criteria and concluded that genetic risk factors predispose to two broad groups of internalizing and externalizing disorders. In another instance, Wright et al. [2] compared the fit of categorical, continuous, and hybrid (i.e., combined categorical and continuous) models of syndromes in an adult epidemiological sample (N = 8841), assessed via structured clinical interviews, and found that the best fitting higher-order model of these syndromes grouped them into three broad spectra: internalizing, externalizing, and psychotic experiences. In turn, Lahey et al. [3] examined the structure of psychopathology—also assessed using structured interviews—in an epidemiological sample of individuals aged 18–65 and found that a bi-factor model in which every mental disorder loaded in a general factor, in addition to the externalizing, distress, and fears factors, presented the best fit for the data. That is, whereas each of the three group factors (externalizing, distress, and fears) accounted well for the correlations among the specific mental disorders that loaded most strongly on those factors, a general dimension captured what all the examined disorders shared in common. Similarly, Caspi and colleagues [4] explored the structure of psychopathology in a longitudinal study that repeatedly assessed individuals from a birth cohort at 18, 21, 26, 32, and 38 years of age, and concluded that psychiatric disorders (assessed via structured interviews) could be explained by three higher-order factors (internalizing, externalizing, and thought disorders), but also found evidence supporting a bi-factor model with one general overriding dimension—termed the *“p* factor”—that captured individuals’ propensity to develop any and all forms of common psychopathologies over and above individual dimensions for each psychiatric disorder, and the three higher-order factors.

Studies in epidemiological samples of children and adolescents have yielded similar results. For example, in a large population-based sample of young people (N = 18,222), Goodman, Lamping, and Ploubidis [5] used confirmatory factor analysis (CFA) to compare the relative fit of three alternative factor structures for the Strengths and Difficulties Questionnaire (SDQ) [6, 7]. The authors concluded that a second-order model with internalizing and externalizing factors (along with a prosocial subscale factor) fitted the data better than a first-order model with the five hypothesized SDQ subscales. These two models had a substantially better fit than the third alternative: a first-order model with internalizing, externalizing, and prosocial factors. These results replicated the classical internalizing-externalizing approach to the structure of child psychopathology [8]. However, Goodman et al. [5] discussed that, while this factorial solution is probably justified in low-risk samples, a five-factor model might be more appropriate in high-risk populations and clinical samples. Yet, this remains to be tested.

It is argued that “lumping” of psychiatric symptoms into broader dimensions can generate models with several advantages. First, they seem to conform to the genetic architecture of psychiatric symptoms given that individual and aggregate molecular genetic risk factors have been found to be shared among a range of psychiatric disorders that are treated as distinct categories in clinical practice [9]. Second, such a parsimonious model of psychopathology could account for the high rates of comorbidity observed among individuals with mental disorders [10]. However, the above-mentioned findings are based on epidemiological samples which, while offering the benefit of being unbiased by referral practices and being generally larger than clinical samples, may not reflect what is seen in clinical practice.

Indeed, in the rare instance where psychiatric symptoms have been analyzed in clinical samples, it has not been possible to group the data in such a reduced number of dimensions. For example, in adults, Kotov et al. [11] found that the best-fitting model for their sample of 2900 outpatients seeking psychiatric treatment was a five-factor solution, including internalizing, externalizing, thought disorder, somatoform, and antagonism dimensions, which fit the data better than a seven-factor model based on the DSM-IV, an internalizing-externalizing model, a three-factor model with an additional somatoform dimension, and an alternative four-factor model which included the previous three dimensions and additionally placed the psychosis, manic episode, and cluster A traits from the internalizing group into a thought disorder dimension. In a sample of German children and adolescents, Becker et al. [12, 13] subjected the items of the SDQ to CFA, and demonstrated a good fit of the original five-factor model both for the parent (N = 543) and the self-reported (N = 214) measure. Interestingly, they also employed exploratory factor analysis (EFA) and the results highly converged with the five original SDQ subscales. The differing evidence between epidemiological and the few studied clinical samples is a clear example of how factorial structure is determined by the type of sample (i.e., the number of ill individuals that these contain and the types of problems that they have) [14], and in this specific case, it suggests that less parsimonious dimensional structures may better reflect the reality of individuals with psychiatric disorders.

In fact, models based on “splitting”—rather than lumping—also have considerable support, particularly in relation to clinical variables. For example, whilst genetic etiology may be largely shared between various anxiety disorders, their distinction may be important in relation to family history, neurobiology, and treatment response [15]. Perhaps even more strikingly, whilst many of the most parsimonious models of psychopathology would consider hyperactivity and conduct problems or irritability as a joint entity, their distinction has key implications for treatment and course. Attention-deficit/hyperactivity disorder (ADHD) symptoms do not respond to parenting interventions [16], whilst conduct and oppositional problems do [16, 17]; conversely, stimulants show large effect sizes particularly for hyperactivity, impulsivity, and inattention, yet less so for irritability and related behaviors [18, 19]. It is therefore crucial to examine whether the structure of psychopathology found in epidemiological samples applies to clinical samples.

Additionally, it remains a matter of debate whether distinct structures of symptoms can have an impact in the prediction of psychiatric outcomes, and whether this is influenced by the type of sample. Results from epidemiological samples have yielded a moderate to high level of agreement between SDQ-generated diagnoses and corresponding clinical diagnoses [20, 21]. More recently, Goodman et al. [5] found that a second-order structure of the SDQ with internalizing, externalizing, and prosocial factors showed clear convergent and discriminant validity when predicting clinical disorders even at the lowest SDQ scores. By contrast, the five SDQ subscales only showed convergent and discriminant validity in children with high scores on those subscales, especially for behavioral and hyperactivity problems. These findings would also support the hypotheses that less parsimonious models account for symptoms in clinical samples.

Results of predictive validity in clinical samples are mixed. Becker and colleagues [12, 13] showed that the total difficulties score of the SDQ was a good predictor of any axis I diagnosis; furthermore, they found that the subscales of the SDQ predicted well their matching diagnostic categories in their clinical sample. However, in a more recent study by Brøndbo et al. [22] on a Norwegian clinical sample, the SDQ was considered insufficient for clinical purposes. The authors also concluded that the SDQ was better in detecting the presence of “any diagnosis” rather than more specific ones and, conversely, was better at ruling out specific diagnoses rather than “any diagnosis.” Given these results, larger clinical samples might be needed to ascertain the predictive value of distinct symptom structures.

While previous studies have examined a first-order five-factor model in young clinical samples [12, 13], no studies to date have offered a comparison of alternative models described in the literature. In addition, no studies in clinical samples have provided with a cross-country validation of these models, hence limiting the generalizability of previous results. Moreover, while several studies have examined the prediction of psychiatric disorders using different symptoms dimensions, no studies have tested whether these predictions hold when these disorders co-occur. The current study tries to fill these gaps in the literature with the following three aims.

First, using CFA in two independent clinical samples from England and Norway, we examine the relative fit of key alternative models using the SDQ, which is one of the most widely-used instruments to measure child and adolescent psychopathology worldwide [e.g., 23, 24]. For comparability, we test the models that have been comprehensively tested before in large epidemiological samples [3–5]. These include a first-order five-factor model, a second-order model with the widely-established broad symptom dimensions of internalizing-externalizing, and two bi-factor models capturing a general psychopathology factor.

Second, as we employ two large samples from different countries, we examine the measurement invariance of the best fitting model across countries to see whether the same structure is generalizable. This is particularly relevant since differences have been found in the presentation of psychopathological symptoms between these countries [25].

Third, we test the external validity of the dimensions. In particular, we test whether each dimension of symptoms—either first- or second-order dimensions—specifically links with psychiatric disorders. Finally, given that comorbidity is typical, we test whether psychopathological symptoms are differently distributed in participants with distinct comorbidities across both samples.

## Methods

### Study Setting and Samples

A clinical sample from London, England (hereafter the England sample) was obtained from the South London and Maudsley (SLaM) NHS Foundation Trust. SLaM is Europe’s largest specialist mental health care area. It serves a population of approximately 1.2 million residents of four South London local authorities. The SLaM Biomedical Research Centre (SLaM BRC) Case Register is a data resource containing de-identified electronic records of all secondary and tertiary mental healthcare service users from SLaM. The Clinical Record Interactive Search (CRIS) is a program which automatically and effectively de-identifies full clinical records derived from the electronic clinical records system in SLaM [26], enabling researchers to search and retrieve de-identified data from these electronic medical records which include over 180,000 cases. Of these, approximately 35,000 are receiving active care from SLaM at a given time. The protocol for this case register has been described in detail elsewhere [27]. CRIS has been used in numerous research studies [e.g., 28–30]. For this study, the variable that defined the main cohort was the existence of an SDQ. Other relevant variables were retrieved for the cohort of participants, including month and year of birth (full dates of birth and postcodes were not available to avoid identification of the individuals), gender, and scores on the Development and Well-Being Assessment (DAWBA), if available. These variables are routinely recorded on SLaM electronic patient records in designated fields. CRIS was approved as a dataset for secondary analysis by Oxfordshire Research Ethics Committee C (reference 08/H0606/71). Approval for the present study was sought and obtained from the CRIS Oversight Committee.

The second large clinical sample, from the University Hospital of North Norway (UNN), Norway (hereafter the Norway sample), includes patients from the Child and Adolescent Mental Health Services (CAMHS) at UNN. UNN is a specialist health area serving a population of 190,726 residents of the county municipalities of Troms and the northern part of Nordland. The health trust is covering an area of approximately 31,300 km^2^. Annually, UNN provides mental health services to about 5% (2100/42,000) of the population aged 0–18 years. About 60% of the treated patients are new referrals from general practitioners and the child protection services. The CAMHS at UNN consist of six outpatient and one inpatient clinics. All of them include the online version of the DAWBA in the routine clinical assessment. The questionnaire SDQ is an integrated part of DAWBA. All DAWBA data at UNN are stored in a de-identified local CAMHS quality register. The data protection officer at UNN has approved the use of data from the quality register for research purposes.

Main characteristics of both the England and the Norway samples, including sample sizes, main demographics, and clinical characteristics, can be found in Table 1. In both sites, the cohorts were defined by the presence of an SDQ, independently of the respondent (parent, teacher, or the young patients themselves). However, only the parent-reported SDQ was used in the analyses. The completion rate of the parent-reported SDQ was 81% (n = 6846) in the England sample and 84% (n = 4940) in the Norway sample. Compared to participants without parent-reported SDQ, those participants with completed parent SDQs were older and more likely to be males in both the England (age: t(5854) = − 18.10, p < 0.0001; gender: χ^2^ = 70.85, p < 0.001) and the Norway sample (age: t(8409) = − 27.63, p < 0.0001; gender: χ^2^ = 86.29, p < 0.001).

**Table 1:** Demographic and clinical characteristics of the South London and Maudsley NHS Foundation Trust and the University Hospital of North Norway samples

	England (N = 8434)		Norway (N = 5866)		Statistics		
	Mean	Sd	Mean	Sd	t	df	*p*
Age (years)^a^	11.2	3.8	12.1	3.6	14.5	14,265	< .0001
SDQ scores^b^							
SDQ total score	18.3	7.7	16.3	6.6	14.6	11,871	< .0001
SDQ emotional	4.7	2.8	4.4	2.7	6.8	11,868	< .0001
SDQ behavioral	3.9	2.6	3.1	2.1	17.8	11,868	< .0001
SDQ hyperactivity	6.2	2.9	5.5	2.8	12.8	11,837	< .0001
SDQ peer problem	3.6	2.4	3.3	2.4	5.1	11,854	< .0001
SDQ prosocial	6.5	2.6	6.9	2.2	7.5	11,868	< .0001
SDQ impact	4.4	2.9	4.4	2.6	1.4	10,418	.15

	n	%	n	%	χ^2^	df	*p*
Gender (females)	3673	44	2802	48	24.1	1	< .001
DAWBA diagnoses^c^							
Emotional disorder^d^	186	77	2116	48	77.6	1	< .001
Behavioral disorder^e^	150	62	2209	51	11.5	1	< .005
ADHD	101	46	1627	37	6.9	1	< .01
ASD	31	16	189	4	54.8	1	< .001

^a^Age range for the England sample 2–18 years; age range for the Norway sample 2–19 years

^b^Number of observations in SDQ scores varies depending on the SDQ subscale (England: 6789–6933; Norway: 4915–4961). Range in SDQ Total Difficulties score is 0–40; range in SDQ subscales is 0–10.\

^c^All disorders are defined with DAWBA bands (> 2). Information about diagnoses was available for 3% of the England sample and 80% of the Norway sample

^d^Any depressive or anxiety disorder

^e^Conduct disorder or oppositional defiant disorder

Participants with missing information on DAWBA diagnoses ranged from 25–27% in the Norway sample and from 97–98% in the England sample (missing values vary across diagnoses). Those with missing information in diagnoses were more likely to be males and older in the Norway sample; whereas these were younger in the England sample.

### Measures

The SDQ is a 25-item questionnaire with five hypothesized subscales: emotional problems, peer problems, behavioral problems, hyperactivity, and prosocial behavior, as well as an additional impact scale [6, 7, 31]. Each subscale comprises five questions with three-point response scales (‘Not true’ = 0, ‘Somewhat true’ = 1, ‘Certainly true’ = 2), with a subscale score range of 0–10. Ten of the 25 items are positively worded ‘strengths’; these are reverse-scored if they contribute to the emotional, peer, behavioral, or hyperactivity subscales. The SDQ has been validated in Norwegian samples [22, 32]. See http://www.sdqinfo.org for a full description of measure and items.

The SDQ parent-report was used due to a number of reasons. First, parents are the main informants in psychiatric services for young people. Second, parent reports have shown to be a better predictor of psychiatric outcomes than self-reports [31, 33]. Third, by using parent report, we could enrich the samples by including children aged 10 years or younger in the analyses, as self-report versions of the SDQ are not available for these ages.

The DAWBA [34] is a detailed psychiatric interview administered by lay interviewers to parents and youth, with a briefer questionnaire for teachers. Each section begins with structured questions that cover the operationalized diagnostic criteria for DSM-IV [35]. Structured questions are supplemented by open-ended questions which record verbatim a respondent’s own description of problem areas. Clinicians review the close and open responses from all informants, identifying discrepancies within or between informants, and using the content, length, and tone of the transcripts to interpret conflicting information [36]. On this basis, raters decide whether a particular child meets all the relevant DSM-IV criteria for an operationalized mental disorder. Raters can also assign ‘Not Otherwise Specified’ disorder, for example ‘behavioral disorder, not otherwise specified’ when children have substantial impairment from symptoms which do not quite meet operationalized criteria. In this paper, we group the mental disorders into emotional disorders (including anxiety and depressive disorders); behavioral disorders (including oppositional defiant and conduct disorders); ADHD; and autism spectrum disorders (ASD; including autism and Asperger’s syndrome). Participants were assigned a positive diagnosis if they scored 3 or higher in the relevant DAWBA bands [37], as previously described [38]. In British samples, the DAWBA has shown to have good inter-rater reliability (e.g., kappa = 0.86 for inter-rater agreement for ‘any mental disorder’ in an epidemiological sample) [39]. It also has good validity as judged against case-notes diagnoses, performs well in differentiating clinic/community samples, and shows strong associations with risk factors, service use, and 3-year prognosis [20, 39]. See http://www.dawba.com for additional information about the DAWBA.

### Statistical Analyses

#### Confirmatory Factor Analyses

**Fig. 1:**
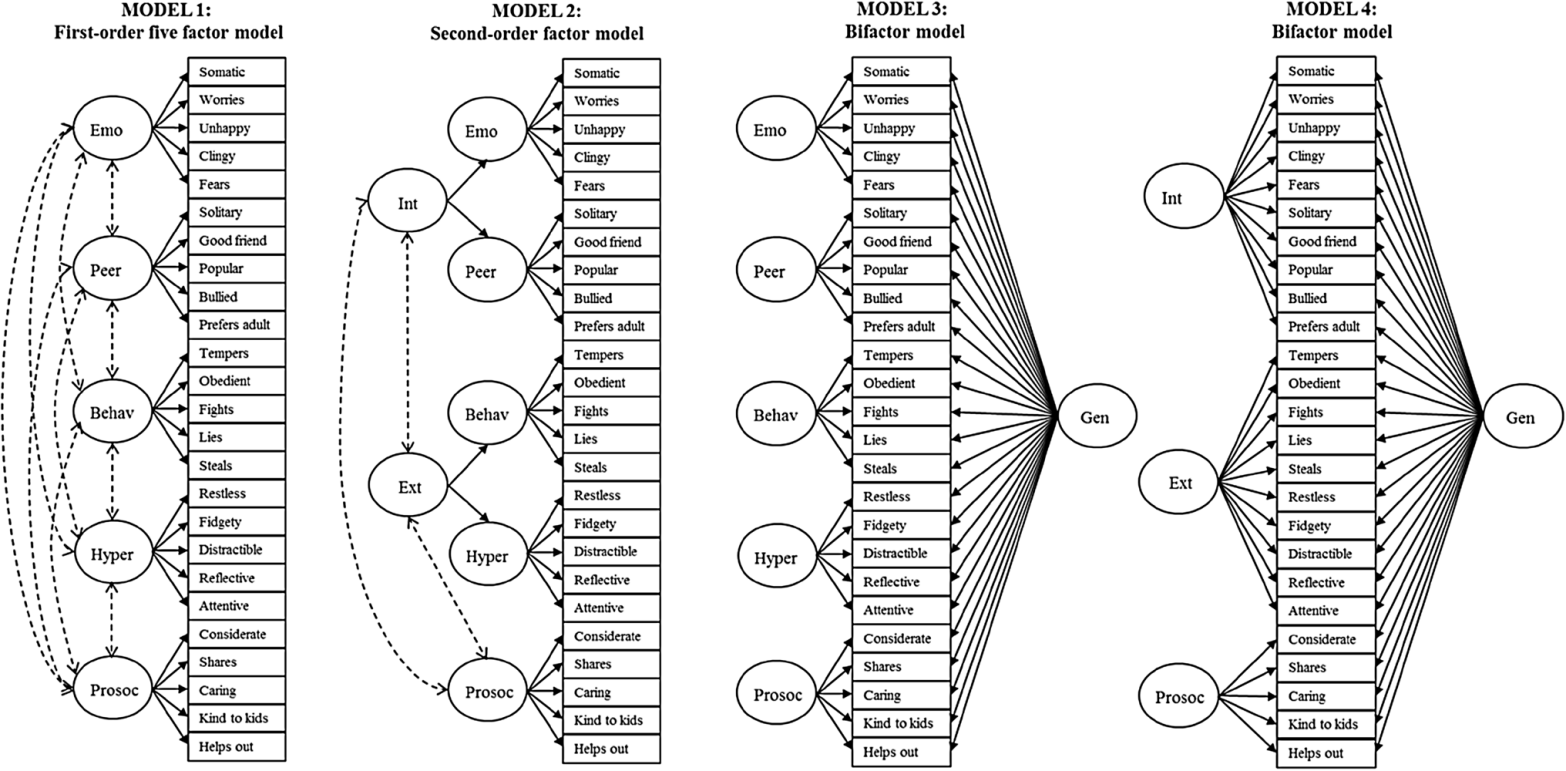
Models to be tested in the confirmatory factor analyses. Solid lines represent factor loadings. Dashed lines represent correlations. *Behav* behavioral problems, *Emo* emotional problems, *Ext* externalizing, *Gen* general factor, *Hyper* hyperactivity problems, *Int* internalizing, *Peer* peer problems, *Prosoc* prosocial problems

We used CFA to evaluate and compare the relative fit of four alternative factor structures for the parent SDQs in the England and Norway samples. These were: (1) a first-order model with the correlated five hypothesized SDQ factors (Model 1) [6]; (2) a second-order model with the SDQ emotional problems and peer problems subscales loading on an ‘internalizing’ factor, plus the SDQ hyperactivity and behavioral problems subscales loading on an ‘externalizing’ factor, with both second-order factors correlating with a first-order factor composed by the items of the SDQ prosocial behavior subscale (Model 2); (3) a bi-factor model including a single general factor and the five SDQ subscales as group factors (Model 3); and (4) a bi-factor model including a single general factor and an externalizing, an internalizing, and a prosocial behavior factor as group factors (Model 4; see Fig. 1). Models 1 and 2 have shown to best fit the data in epidemiological samples using the same instrument [5], whereas Models 3 and 4 examine the existence of a general psychopathological factor similar, though not identical, to that found previously [3, 4].We performed the CFA in MPlus version 5.1, using a multivariate probit analysis for ordinal data [40, 41] and estimating model fit using the Weighted Least Squares, Mean, and Variance adjusted (WLSMV) estimator. We followed common practice in reporting multiple indices of fit, namely the Comparative Fit Index (CFI), the Tucker Lewis Index (TLI), and the Root Mean Square Error of Approximation (RMSEA) [42, 43]. To consider a model as showing ‘acceptable’ fit, we required a CFI > 0.90, TLI > 0.90, and RMSEA < 0.08; to consider a model as showing ‘good’ fit, we required a CFI > 0.95, TLI > 0.95, and RMSEA < 0.06 [43]. χ^2^ difference test was used to compare nested models, where improvements in model fit by the nested—less constrained and more parsimonious—model are tested. We also compared models using the Akaike Information Criteria (AIC) and the Bayesian Information Criteria (BIC), where lower values indicate better fit [44, 45]. Unlike χ^2^, AIC and BIC penalize per increasing number of estimated parameters, thus avoiding overfitting models. Where models showed acceptable fit on some indices but not on others, we allowed correlations between the unique variances of some individual items within the same factor, selecting these item pairs using MPlus’ modification indices. Such minor model modifications can improve model fit by increasing the proportion of variance explained, but do not change the substantive conclusions regarding the adequacy of a hypothesized factor structure in describing a set of data [46].

#### Measurement Invariance

Measurement invariance (MI) is present when a specific instrument (e.g., SDQ) measures the same construct across different groups [47]. If MI is established, then it can be confirmed that the participants across all groups interpret the individual items, as well as the underlying latent factors, in the same way. This is particularly important in cross-cultural research when comparing results across different cultures and people speaking different languages (in this case, English and Norwegian). Conversely, failure to prove MI indicates that groups interpret the items differently and, as a consequence, factor means cannot be compared in a meaningful way [48, 49].

Here we tested MI across gender (males vs. females), groups of age (2 to 11 year-old children vs. 12 to 18 year-old adolescents), and countries (England vs. Norway) to examine whether the results from the CFA were consistent across these groups and allow comparisons between the two different samples included in the study. MI was tested in MPlus version 5.1 using WLSMV estimator, and theta parameterization.

MI examines the change in the goodness-of-fit indices (GFI) when cross-group constraints are imposed on a measurement model in a hierarchical set of steps [50, 51]. Configural, metric, scalar, and measurement error invariance were tested. Configural invariance refers to whether the same CFA is valid in each group. Metric invariance—also called weak factorial invariance—concerns the equivalence of the factorial loadings across groups. Scalar (or strong factorial) invariance is assumed when the item intercepts and the factor loadings are equally constrained across groups. Finally, when testing measurement error (or strict factorial) invariance, also the variances of the residuals are constrained across groups.

The Chi square difference test is widely used to compare nested models [52]. However, this test yields significant differences in large sample sizes [53]. Therefore, we compared models on the basis of changes in CFI (ΔCFI) as suggested by Cheung and Rensvold [47]. A value of ΔCFI higher than or equal to − 0.01 indicates that the null hypothesis of invariance should not be rejected.

#### Predictive Value of the SDQ Subscales

We performed a series of logistic regression analyses in Stata 11 using DAWBA diagnoses for any emotional disorder, any behavioral disorder, ADHD, or ASD as outcomes. All five hypothesized SDQ subscales were entered in the model as predictors of interest. In a second step, we employed internalizing and externalizing problems as predictors as a comparison. Given the high rates of missing values in DAWBA diagnoses in the England sample, we replicated these analyses in MPlus using Full Information Maximum Likelihood (FIML). Maximum likelihood uses all the available data to generate parameter estimates without the need to discard cases or fill in the data prior to analysis. This method is currently regarded as the “state of the art” missing data technique [54] given that requires of less strict assumptions about the mechanism that led to missing data and generally produces more accurate estimates than traditional missing data handling techniques (e.g., discarding cases).

In addition, given that comorbidity was frequent across both samples (56% of participants with a diagnosis met criteria for at least a second diagnosis), we tested whether SDQ subscales were differently distributed among groups of comorbidity. We focused these analyses in participants with two diagnoses, who represented 63% of those with comorbidities. We performed comparisons across three different groups: emotional + conduct disorder (CD) (n = 443), emotional + ADHD (n = 123), and CD + ADHD (n = 524). Since rates of ASD were low compared to other disorders (n = 14 CD + ASD; n = 12 emotional + ASD; and n = 5 ADHD + ASD), comorbid ASD groups were excluded from the analyses. Levene’s robust test statistic (W0) for the equality of variances showed that variance of SDQ emotional, peer problems and prosocial subscale scores did not differ across comorbidity groups (all p > 0.300), and these were examined with analysis of variance (ANOVA) with Bonferroni correction. Variances of SDQ behavioral (W0 = 7.98, p < 0.001) and hyperactivity (W0 = 17.33, p < 0.001) subscales scores differed across groups, and these were examined with Welch ANOVA with Bonferroni correction.

In all analyses, we reverse-scored the SDQ prosocial subscale in order to facilitate comparisons of effect sizes across subscales.

## Results

### Confirmatory Factor Analyses

**Fig. 2:**
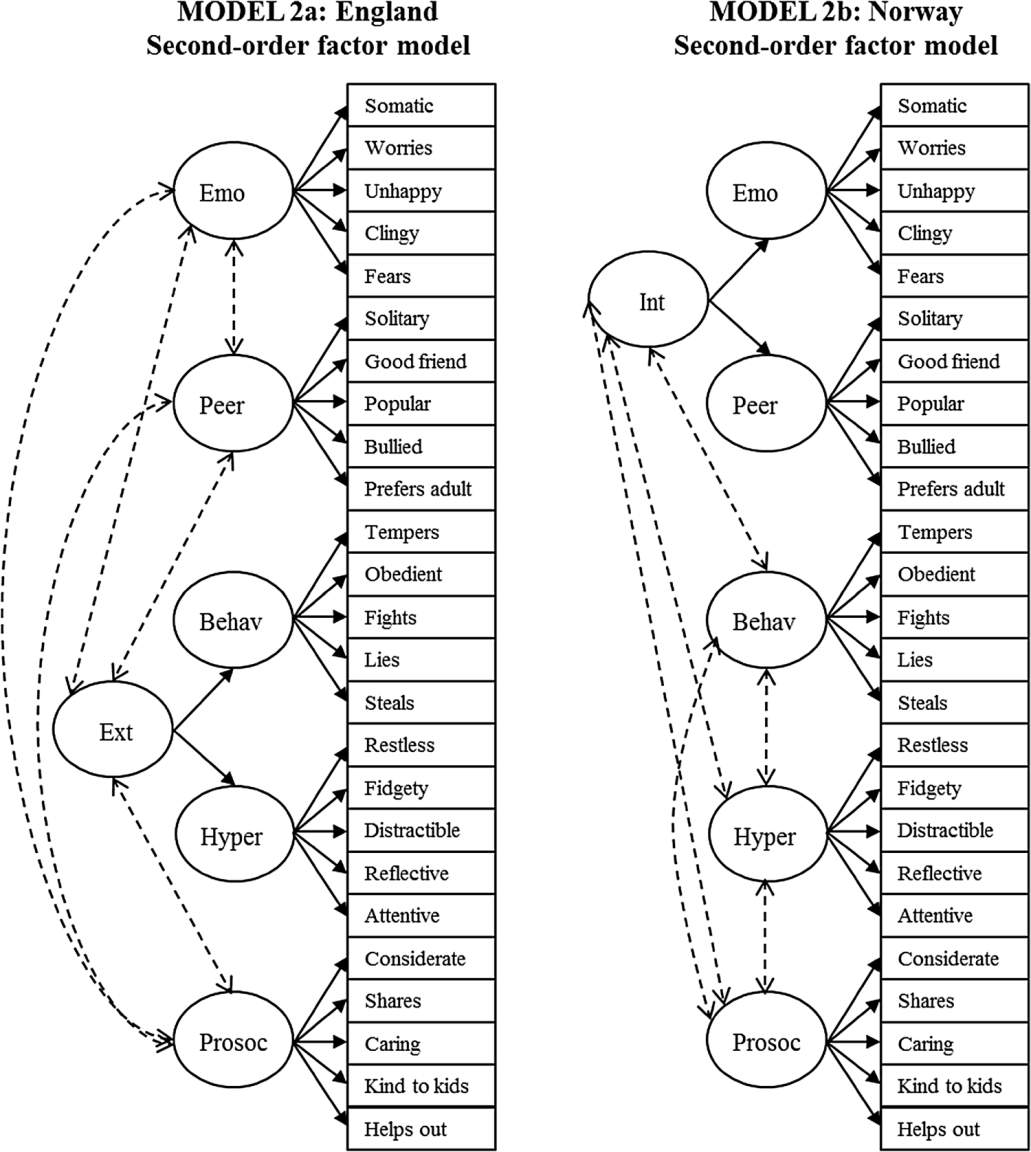
Second-order factor models used in the confirmatory factor analyses. Solid lines represent factor loadings. Dashed lines represent correlations. *Behav* behavioral problems, *Emo* emotional problems, *Ext* externalizing, *Hyper* hyperactivity problems, *Int* internalizing, *Peer* peer problems, *Prosoc* prosocial problems

The first-order five-factor model (Model 1) converged well. However, the second-order factor model (Model 2) yielded Heywood cases in both samples (i.e., negative residual variances and correlations greater than 1.00). Specifically, in the England sample, the first-order factor ‘peer’ showed a negative residual variance and a standardized association greater than 1.00 with the second-order factor ‘internalizing’. In contrast, in the Norway sample, the factor ‘behavioral’ showed the same pattern with the ‘externalizing’ factor. These findings indicate an overlapping of variance between the first- and second-order factors such that discriminant validity between these two constructs is indistinguishable. In other words, the second-order factor predicts almost perfectly one of the first-order factors [55]. Given these results, we modified the models as shown in Fig. 2 (Model 2 modified to Models 2a and 2b for the England and Norway sample, respectively).

Model 3 (five group factors and a general bi-factor) only converged in the England sample and showed that the variance of the general factor was very low (0.011). In addition, the standardized loadings of the emotional items into the general factor were also low, ranging from 0.07 to 0.27. In order to make Model 3 converge in the Norway sample, as well as Model 4 in both samples, we had to fix variances of all factors at 1 and free the estimation of the first indicator. In all cases, the standardized loadings of the emotional items into the general factor were low, ranging from 0.00 to 0.31.

As seen in Table 2, the five hypothesized SDQ factors (Model 1) and the second-order factor model with additional ‘internalizing’ or ‘externalizing’ factors (Models 2a and 2b), showed acceptable fit in all indices (CFI > 0.85, TLI > 0.90, RMSEA < 0.08). In contrast, both bi-factor models only showed acceptable fit in CFI and RMSEA in the England sample.

**Table 2:** Model fit in confirmatory factor analyses of the England and Norway samples’ strengths and difficulties questionnaires (SDQs)

Sample	Model^a^	χ^2^	df	*p*	CFI	TLI	RMSEA
England^b^ N = 6912	Model 1	7517.16	256	< 0.0001	0.923	0.909	0.064
	Model 2a	7367.15	258	< 0.0001	0.924	0.912	0.063
	Model 3	9082.67	250	< 0.0001	0.906	0.887	0.071
	Model 4^d^	8460.17	250	< 0.0001	0.912	0.895	0.069
Norway^c^ N = 4961	Model 1	6104.11	254	< 0.0001	0.921	0.907	0.068
	Model 2b	6089.42	256	< 0.0001	0.922	0.908	0.068
	Model 3^d^	8446.68	250	< 0.0001	0.890	0.868	0.081
	Model 4^d^	9533.07	250	< 0.0001	0.875	0.850	0.087

^a^Model 1 is displayed in Fig. 1; Models 2a, and 2b are displayed in Fig. 2

^b^England sample minor modifications: allowing correlation between the unique variance of (clingy and unhappy) (steals and lies) (fidgety and restless) (reflective and restless) (reflective and fidgety) (persistent and restless) (persistent and fidgety) (persistent and distractible) (volunteers and helpful)

^c^Norway sample minor modifications: allowing correlation between the unique variance of (clingy and fears) (clingy and unhappy) (steals and lies) (distractible and restless) (distractible and fidgety) (restless and fidgety) (reflective and fidgety) (reflective and restless) (persistent and restless) (persistent and fidgety) (persistent and distractible)

^d^Factor variances had to be fixed at 1 to make the model converge

Comparison of nested Models 1 and 2 yielded significant χ2 difference tests (England: Δχ2 = 17.74; Δdf = 2; p = 0.0001. Norway: Δχ2 = 73.39; Δdf = 2; p < 0.0001), favoring Model 1 (five first-order factors) compared to Models 2a and 2b across both samples. However, in the comparison of bi-factor models (Models 3 and 4) with Models 1 and 2, computation of Δχ^2^ was not possible due to the singular matrix during the computation process.

Overall, and taking all the goodness-of-fit indices into account, Model 1 (first-order five-factor solution) was the one that showed the best fit to the data among all the models tested.

### Measurement Invariance

In order to test whether our results were comparable across gender, age, and site, we tested the MI. Overall, and in line with our hypothesis, MI held for all comparisons by gender, age, and country for the model that had shown the best fit in the previous analyses (Model 1).

In all cases, ΔCFI was equal to or higher than − 0.01 (range = − 0.010 to 0.017) when compared to the preceding model, which suggests that the model should not be rejected (Table 3).

**Table 3:** Fit indices for invariance test of the five-factor model

Sample/model	χ^2^	df	*p*	CFI	TLI	RMSEA	ΔCFI
England							
Gender: males (n = 4066) versus females (n = 2844)							
Configural invariance	5560.73	279	< 0.0001	0.879	0.934	0.074	–
Metric invariance	5819.13	295	< 0.0001	0.874	0.935	0.074	− 0.005
Scalar invariance	5841.17	304	< 0.0001	0.873	0.937	0.073	− 0.001
Error variance invariance	5082.82	289	< 0.0001	0.890	0.942	0.069	0.017
*Age*: 2–11 years old (n = 3864) versus 12–18 years old (n = 3048)							
Configural invariance	5967.37	276	< 0.0001	0.876	0.934	0.077	–
Metric invariance	6160.22	290	< 0.0001	0.872	0.935	0.077	− 0.004
Scalar invariance	6531.73	299	< 0.0001	0.864	0.933	0.078	− 0.008
Error variance invariance	5818.53	285	< 0.0001	0.879	0.938	0.075	0.015
Norway							
Gender: males (n = 2708) versus females (n = 2253)							
Configural invariance	4196.60	262	< 0.0001	0.897	0.934	0.078	–
Metric invariance	4396.89	274	< 0.0001	0.892	0.934	0.078	− 0.005
Scalar invariance	4410.24	281	< 0.0001	0.892	0.936	0.077	0.000
Error variance invariance	4085.97	278	< 0.0001	0.901	0.940	0.074	0.009
Age: 2–11 years old (n = 2245) versus 12–18 years old (n = 2716)							
Configural invariance	4475.85	260	< 0.0001	0.887	0.929	0.081	–
Metric invariance	4759.46	272	< 0.0001	0.880	0.928	0.082	− 0.007
Scalar invariance	5018.10	280	< 0.0001	0.873	0.926	0.083	− 0.007
Error variance invariance	4859.67	277	< 0.0001	0.878	0.928	0.082	0.005
England and Norway							
Country: England (n = 6912) versus Norway (n = 4961)							
Configural invariance	9855.78	273	< 0.0001	0.889	0.936	0.077	–
Metric invariance	10442.59	287	< 0.0001	0.883	0.935	0.077	− 0.006
Scalar invariance	11291.82	296	< 0.0001	0.873	0.932	0.079	− 0.010
Error variance invariance	11359.88	289	< 0.0001	0.872	0.930	0.080	− 0.001

### Predictive Value of the SDQ Subscales

Finally, we wanted to test whether each dimension of symptoms of the SDQ specifically linked with diagnostic outcomes. The results in the invariance testing allowed us to merge both clinical samples in order to predict clinical diagnoses.

Table 4 shows which subscales (including internalizing and externalizing scores) had the largest effect on the odds of receiving a DAWBA diagnosis. In all cases, the expected subscale(s) always had the largest point estimates of effect size. These point estimates were also substantially and significantly larger than the next-largest estimates, except for the peer and prosocial subscales predicting ASD, which were not significantly different from the hyperactivity or externalizing subscales. The prediction of emotional and behavioral disorders was stronger using the five-subscale model as evidenced by the non-overlap of the 95% confidence interval estimates with the three-subscale model (emotional: t = 3.22, p = 0.0013; behavioral: t = 4.03, p = 0.0001). The analyses using FIML, which included all the available data (N = 11,918–11,925), replicated the previous results; i.e., emotional disorder was best predicted by SDQ emotional score (OR = 3.65, p < 0.001), CD was best predicted by SDQ behavioral score (OR = 6,90, p < 0.001), ADHD was best predicted by SDQ hyperactivity score (OR = 6,64, p < 0.001), and ASD was best predicted by SDQ peer problems and prosocial scores (OR = 2.48 and 2.00, respectively, both p < 0.001).

**Table 4:**
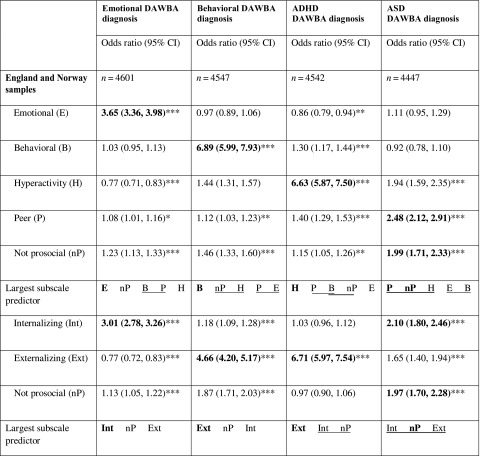
Association of the strengths and difficulties questionnaire (SDQ) subscales with the development and well-being assessment (DAWBA) diagnoses at baseline

*p < 0.05, **p < 0.01, ***p < 0.001. Odds ratios presented for probability of DAWBA diagnosis per one standard deviation increase in the SDQ subscale in question. Results in bold are the hypothesized association for the disorder in question. Below the odds ratios, the five subscales are presented in order of magnitude; subscales sharing an underline were not significantly different at p < 0.05. Note that the prosocial score is reverse-scored to facilitate comparisons of effect sizes. All subscales are entered in the model as predictors. N is the number of observations included in the model

**Fig. 3:**
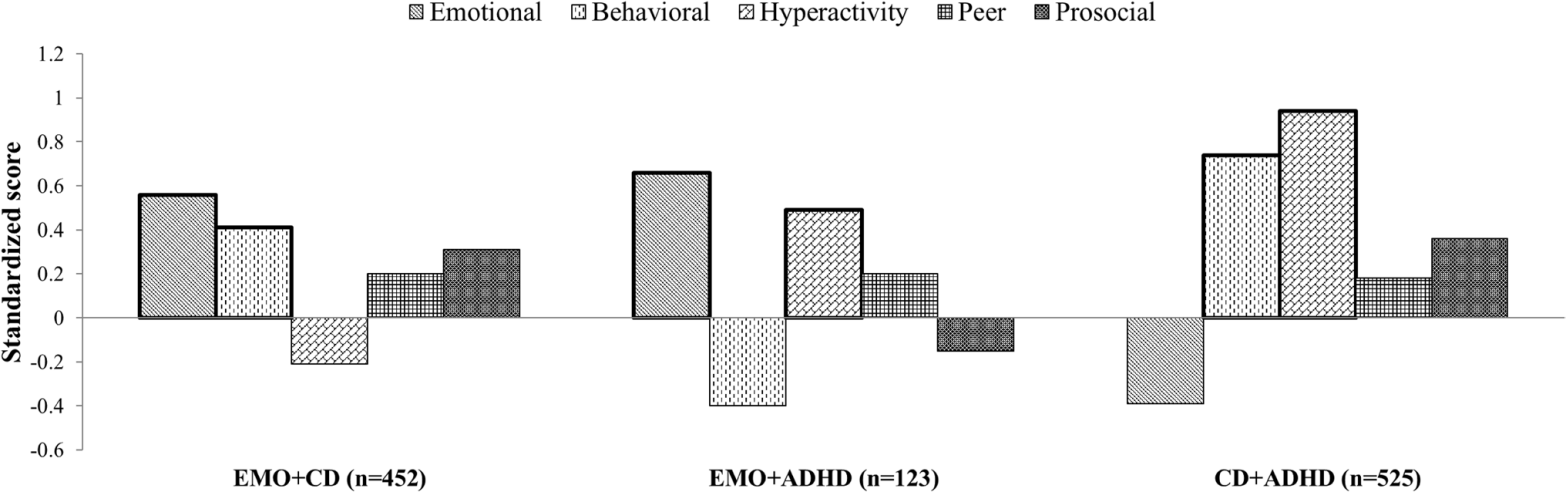
Standardized SDQ subscale scores across different comorbidities. Bars with bold border are those scores hypothesized to be higher for that group. *ADHD* attention-deficit/hyperactivity disorder, *CD* conduct disorder, *EMO* emotional disorders

Analyses of variance with those participants with comorbidities showed a pattern similar to that obtained using the logistic regression analyses. Scores of SDQ emotional subscale were higher in the two groups with emotional disorder compared to the CD + ADHD (all p values < 0.001). SDQ behavioral subscale scores were higher in the two groups with CD compared to Emotional + ADHD (all p values < 0.001), as well as higher in CD + ADHD than in CD + Emotional (p < 0.001). SDQ hyperactivity scores were higher in the two groups with ADHD, compared to the group with CD + Emotional (all p < 0.001). CD + ADHD also scored higher in hyperactivity than the emotional + ADHD group (p < 0.001). There were no differences in SDQ peer problems between groups. Reversed SDQ prosocial scores were higher in the two groups with CD compared with Emotional + ADHD (all p values < 0.001). Standardized SDQ subscale scores for each comorbidity group are depicted in Fig. 3.

## Discussion

In this study, we used data from 14,209 children and adolescents from clinical settings (8343 from England and 5866 from Norway) to explore the symptom structure of their psychiatric symptoms using a widely used psychometric tool, the SDQ. Results showed that a five-factor structure presented the best fit for the data in both samples and was superior to second order factor models with additional ‘internalizing’ and ‘externalizing’ factors, as well as to two bi-factor models that accounted for a general factor. This finding contrasts with those of previous epidemiological studies [e.g., 5], suggesting that psychiatric disorders present with unique phenotypic characteristics that need to be taken into account, and that a too simplified approach may not be appropriate when dealing with patients in real-world settings. As reported by Goodman et al. [5], discriminating symptom clusters may be easier when focusing on children with more severe mental health problems (i.e., a clinical population) and this differentiation may be more difficult to establish when levels of psychopathology are low (like in epidemiological samples). It is possible that the expression, perception, and report of symptoms in low risk samples might be unspecific and blurred, whereas in high-risk samples the specificity of symptoms for each disorder increases. Such a pattern would inevitably influence the factorial structure of symptoms. Additionally, epidemiological studies have generally missed out participants suffering from serious mental health problems or otherwise disadvantaged [56]. In our study, mean SDQ Total Difficulties scores ranged between 16.3 and 18.3. In contrast, mean scores in epidemiological studies never reach these levels of severity, with average scores ranging around 7.5–11.0 points [e.g., 57, 58].

It could be argued that our findings, which contrast with those obtained in epidemiological studies, are due to referral biases in our samples. However, even if such biases were operating, it would not take away from the fact that a substantial proportion of severely impaired young people—those who attend clinics—show a structure of psychopathological symptoms that is different from that observed in epidemiological studies. Moreover, it would be expected that at least some of the referral biases would be different between England and Norway—yet, we demonstrate strict factorial invariance across the two samples. This becomes especially relevant when taking into account that the two countries under study have shown differences in the presentation of their psychiatric symptoms, also measured by the SDQ [e.g., 25, 59]. MI analyses also showed that, at least in clinical samples, psychiatric symptoms cluster in these five factors in boys and girls of different ages. Our results suggest that, given a certain level of severity, groups of symptoms might be already defined from early stages in the development with no distinctions across genders. This would be in line with previous research showing that psychopathology appears to be differentiated among younger children as much as it is among older children [60]. Interestingly, in a recent study across five European countries using an epidemiological sample of adolescents (N = 3012) which also used the SDQ [57], MI across countries was only partial (11 items out of 25 were invariant), suggesting that some items should be considered carefully when using across countries. However, this study used the self-reported version of the questionnaire and participants were older than those in our sample (mean age = 14.20). Authors point out that the developmental changes that occur in adolescence could be different depending on factors such as the geographical area, the culture, and the meaning of the items or the language.

A more granular approach to psychiatric symptoms, where several dimensions of symptoms are considered, also shows to be helpful when predicting psychopathology. In keeping with our hypotheses, the split between SDQ subscales allowed for distinctions between disorders, even when other disorders were also present. This finding is relevant for etiological studies. Twin studies and risk-factor studies have shown that there are substantial phenotypic correlations among pairs of psychiatric disorders that are influenced by the same genetic factors [e.g., 61, 62]. This indicates that causes of the disorders may be similar and seems to encourage a transdiagnostic approach to psychiatric disorders [4]. Additionally, this more parsimonious approach may be helpful when looking at low-risk samples [5] or when studying correlates, neural mechanisms, outcomes that are common across mental disorders [3], and factors of resilience to psychiatric disorder [33]. However, in clinical samples and when looking for models that can inform clinical predictions and treatment choices, a model considering a broader range of symptom dimensions could be more accommodating. The differences in treatment response across seemingly related behaviors/symptoms [16–19] suggest that, in order to understand the genetic or neural substrates of psychopathology, we should probably use more specific models which split patterns of symptoms. Clinicians can benefit from this approach to more sensitively screen patients referred to mental health services. An instrument as short as the SDQ seems to be helpful in making distinctions provided its multi-dimensional structure is retained. However, it is important to note that these multiple dimensions are still grouping together a number of conditions (e.g., the emotional scale of the SDQ may contain a range of anxiety and depressive disorders that may require different treatment approaches) and, hence, an in depth assessment that takes into account the specificity of psychopathology at the diagnostic level is still preferred if time and resources allow.

The results of this study need to be considered in light of its limitations. First, we only used parent-reported measures in our analyses. However, those are typically collected in pediatric populations, especially in younger children. Second, the structure of psychopathology may be, to some extent, influenced by the type of instrument used. Hence, we cannot rule out the possibility that different instruments could lead to different results. Studies in adult studies have used different assessment methods and, hence, a head to head comparison between pediatric and adult samples may be difficult. However, the pediatric studies with which we are comparing our results have also used the SDQ. Third, each section of the DAWBA uses skip-rules, one component of which is in some occasions the relevant SDQ subscale. For example, in the hyperactivity disorder section, parents positively reporting ‘some problems with hyperactivity or poor concentration’ *or* an SDQ hyperactivity score ≥ 6 for their child, will continue responding items in the section; otherwise, they will be directed to the next section. Therefore, predicting DAWBA diagnoses using the SDQ subscale scores might be somewhat circular. However, in order to test to what extent circularity would have affected our results, we performed additional analyses (see Supplemental Material) using the Avon longitudinal study of parents and children (ALSPAC) sample [63], where SDQ skip rules were not employed to define DAWBA diagnoses. The results of these analyses showed that using SDQ skip rules to define diagnoses—against not using these rules—did not modify significantly the predictions. Most importantly, the specificity of predictions between SDQ subscales and relevant diagnoses was clear in both approaches.

## Summary

It has been suggested that the structure of psychiatric disorders can be reduced to a few symptom dimensions. These proposals, mainly based on epidemiological samples, may not apply to clinical populations. We aimed to test the structure of psychiatric symptoms across two pediatric clinical samples from different countries and to ascertain the predictive value of each factor within them. Two clinical samples from England (N = 8434) and Norway (N = 5866)—assessed with the parent-reported SDQ and the DAWBA—were used to test our aims. Confirmatory factor analyses of the parent-reported SDQ evaluated the relative fit of several models, including a first-order model, a second-order model with the widely-established broad symptom dimensions of internalizing-externalizing, and two bi-factor models capturing a general psychopathology factor. Measurement invariance was examined to establish whether both samples were meaningfully comparable. Predictive value of the SDQ subscales for psychiatric disorders was examined through logistic regressions. A first-order five-factor solution better fit the data in both samples. Measurement invariance held for all comparisons by gender, age, and country. In all cases, the expected SDQ subscale(s) related best to the corresponding DAWBA diagnosis (e.g., emotional to emotional disorder; conduct problems to conduct disorder; hyperactivity to ADHD), even in cases of comorbidity. Our findings suggest that, in clinical samples, a more granular approach to psychiatric symptoms where several dimensions are considered seems to fit the data better than a model based alone on splitting between internalizing/externalizing dimensions. This approach is also helpful when predicting psychopathology. Future research needs to examine whether this factor structure holds across other clinical samples and other psychometric measures using a variety of informants (including teachers and the patients themselves).

## Electronic supplementary material

Below is the link to the electronic supplementary material.

Supplementary material 1 (DOCX 33 KB)
