# Supplementary material for: Should Clinicians Split or Lump Psychiatric Symptoms? The Structure of Psychopathology in Two Large Pediatric Clinical Samples from England and Norway
Source: Child Psychiatry Hum Dev. 2017 Dec 14;49(4):607–20. doi: 10.1007/s10578-017-0777-1 (PMC6019426; doi:10.1007/s10578-017-0777-1)
Supplement: Supplementary file 1 — Supplementary material 1 (DOCX 33 KB) [file 10578_2017_777_MOESM1_ESM.docx]

**SUPPLEMENTAL MATERIAL**

Each section of the DAWBA uses skip-rules, one component of which is in some occasions the relevant SDQ subscale. For example, in the hyperactivity disorder section, parents positively reporting ‘some problems with hyperactivity or poor concentration’ or an SDQ hyperactivity score ≥ 6 for their child, will continue responding items in the section; otherwise, they will be directed to the next section. In order to test to what extend circularity between SDQ subscales scores and DAWBA diagnoses would have affected our results, we performed additional analyses using data from the first wave of the Avon longitudinal study of parents and children (ALSPAC) sample [63], where SDQ skip rules were not employed to define DAWBA diagnoses.

We replicated the same validity analysis employing the five SDQ subscales as predictors and the most relevant clinical diagnosis as outcome (i.e., emotional disorder, ADHD, conduct disorder). We first ran the analyses without using the SDQ skipping rules for the definition of DAWBA diagnosis, where circularity is not present. We then repeated the analyses excluding positive cases (e.g., children with emotional disorders) based in the cut-offs of the relevant SDQ subscale; that is, using SDQ skipping rules, where circularity is then present as in our study.

We report here the results of logistic regressions using SDQ subscales as predictors and DAWBA diagnosis as outcomes, before and after using the SDQ skip rules (**Table S1**).

The results in **Table S1** show three things:

1. As expected, odds ratios for the prediction of DAWBA diagnoses slightly increased in some cases when using SDQ skip rules to define these diagnoses.
2. However, the difference in the prediction from the relevant scale is not significantly different. This can be seen by looking at the overlap between 95% confidence interval of the odds ratios.
3. Most importantly, the specificity in the prediction is clear in both approaches.

**Table S1.** Association of the Strengths and Difficulties Questionnaire (SDQ) subscales with the Development and Well-Being Assessment (DAWBA) diagnoses at baseline.

| Circularity ^a^ | | **Emotional DAWBA diagnosis** | **Behavioral DAWBA diagnosis** | **ADHD**  **DAWBA diagnosis** |
| --- | --- | --- | --- | --- |
|  |  | Odds ratio (95%CI) | Odds ratio (95%CI) | Odds ratio (95%CI) |
| **Not present** |  | *n=7633* | *n=7253* | *n=7270* |
|  | Emotional (E) | **1.31 (1.21, 1.41)***** | 1.02 (0.90, 1.16) | 1.00 (0.84, 1.17) |
|  | Behavioral (B) | 1.07 (0.97, 1.17) | **2.28 (1.99, 2.62)***** | 1.29 (1.08, 1.53)** |
|  | Hyperactivity (H) | 1.20 (1.10, 1.31)*** | 1.96 (1.68, 2.28)*** | **5.56 (4.31, 7.18)***** |
|  | Peer (P) | 1.07 (0.99, 1.16) | 1.34 (1.20, 1.50)*** | 1.45 (1.25, 1.68)*** |
|  | Not Prosocial (nP) | 0.94 (0.86, 1.03) | 1.22 (1.06, 1.39)** | 1.17 (0.98, 1.41) |
| *Largest subscale predictor* | | **E**  H P B nP | **B** H P nP E | **H** P B nP E |
| **Present** |  |  |  |  |
|  | Emotional (E) | **1.31 (1.21, 1.41)***** | 1.02 (0.90, 1.16) | 1.00 (0.84, 1.17) |
|  | Behavioral (B) | 1.06 (0.97, 1.16) | **2.40 (2.08, 2.76)***** | 1.29 (1.08, 1.53)** |
|  | Hyperactivity (H) | 1.20 (1.09, 1.31)*** | 1.98 (1.70, 2.32)*** | **5.56 (4.31, 7.18)***** |
|  | Peer (P) | 1.07 (0.99, 1.16) | 1.35 (1.20, 1.51)*** | 1.45 (1.25, 1.68)*** |
|  | Not Prosocial (nP) | 0.94 (0.86, 1.03) | 1.23 (1.07, 1.41)** | 1.17 (0.98, 1.41) |
| *Largest subscale predictor* | | **E**  H P B nP | **B** H P nP E | **H** P B nP E |

a Circularity is defined as the use of SDQ skipping rules to define DAWBA diagnoses.

*p<0.05, **p<0.01, ***p<0.001. Odds ratios presented for probability of DAWBA diagnosis per one standard deviation increase in the SDQ subscale in question. Results in bold are the hypothesized association for the disorder in question. Below the odds ratios, the five subscales are presented in order of magnitude; subscales sharing an underline were not significantly different at p<0.05. Note that the prosocial score is reverse-scored to facilitate comparisons of effect sizes. All subscales are entered in the model as predictors. N is the number of observations included in the model.
